# High prevalence of hepatitis E and rat hepatitis E viruses in wastewater in Gothenburg, Sweden

**DOI:** 10.1016/j.onehlt.2024.100882

**Published:** 2024-08-22

**Authors:** Marianela Patzi Churqui, Margarita Ghaleb, Timur Tunovic, Miriam Frankal, Lucica Enache, Kristina Nyström, Martin Lagging, Hao Wang

**Affiliations:** a Institute of Biomedicine, Department of Infectious Diseases, University of Gothenburg, Gothenburg, Sweden; b Sahlgrenska University Hospital, Department of Clinical Microbiology, Region Västra Götaland, Gothenburg, Sweden; c Faculty of Medicine and Health Sciences, Linköping University, Linköping, Sweden; d Region Västra Götaland, Södra Älvsborg Hospital, Clinic of Infectious Diseases, Borås, Sweden; e Department of Research, Education and Innovation, Region Västra Götaland, Södra Älvsborg Hospital, Borås, Sweden; f Gryaab AB, Gothenburg, Sweden

**Keywords:** Wastewater-based epidemiology, Zoonotic transmission, Surveillance, Sewage, HEV-C, Subtype

## Abstract

Hepatitis E virus (HEV) and Rat Hepatitis E virus (RHEV), recognized for their zoonotic potential, pose significant public health concerns. Our previous research identified both viruses in effluent wastewater in Gothenburg, Sweden. However, there are lingering inquiries regarding the prevalence and genetic diversity of these viruses in influent wastewater, as well as the utility of wastewater surveillance in elucidating their community circulation dynamics. To address these knowledge gaps, we conducted weekly collection of wastewater samples at the Rya wastewater treatment plant in Gothenburg throughout 2023. The concentrations of HEV and RHEV were quantified using quantitative polymerase chain reaction (qPCR). Additionally, two semi/nested-PCR were utilized to amplify viral strains. Furthermore, HEV strains from patients within the same region, as well as other regions in Sweden in 2023, were incorporated into the analysis. Remarkably, we observed a high prevalence of HEV (86%) and RHEV (98%) in wastewater samples, with the majority of HEV sequences identified as subtype 3c/i (9/12). In contrast, HEV subtype 3f was the most sequenced among clinical patient samples (6/12). Notably, previously unreported HEV-3b and unclassified strains were detected in wastewater. Almost all RHEV strains (20/21) were clustered into European groups, with none of the RHEV genetically close to strains previously found in human cases. The notable discordance in prevalence and identified subtypes of HEV-3 in wastewater compared to clinical samples suggests either a significant underdiagnosis of HEV infections or differences in viral loads and shedding durations among humans between HEV-3 subtypes. This underscores the urgent need for improved diagnostic techniques and heightened awareness of HEV transmission dynamics. Furthermore, the consistent detection of RHEV in wastewater underscores the necessity for further investigations to assess the potential role of RHEV in hepatitis cases of unknown etiology, given that most currently available clinical diagnostic assays fail to detect RHEV.

## Highlights

• A high prevalence of HEV and RHEV were identified in wastewater in Gothenburg, Sweden.
• HEV subtype 3c/i dominates in wastewater, whereas 3f prevalent in patients.
• Unreported HEV3b and unclassified strains identified through wastewater monitoring.
• Distinct RHEV clades in wastewater, but not similar to previous human strains.

## 1 Introduction

Members of the *Hepeviridae* family are classified into two subfamilies, *Parahepevirinae* and *Orthohepevirinae*. Within the *Orthohepevirinae* subfamily, there are four genera and nine species [1]. One such species, *Paslahepevirus balayani*, formerly recognized as *Orthohepevirus* A (HEV-A), exhibits the ability to infect humans and various mammalian species [2]. HEV-A encompasses eight genotypes, designated as HEV-1 to HEV-8, with HEV-1 to HEV-4 being predominantly associated with human HEV infections [3]. In Europe, human HEV infections are predominantly attributed to foodborne and zoonotic transmission by HEV-3 [4]. Nonetheless, reports and suspicions of alternative transmission routes for HEV-3 in industrialized countries, including blood transfusion and waterborne transmission, underscore the complexity of HEV transmission and warrant further investigation into additional potential routes of infection [5,6].

Another species within the *Orthohepevirinae* subfamily with the potential to infect humans is *Rocahepevirus ratti*, previously referred as *Orthohepevirus* C (HEV-C), specifically rat hepatitis E virus (RHEV). This species is phylogenetically distinct from HEV-A [2]. RHEV was initially discovered in Germany in 2010 and subsequently identified in rodents, shrews, and carnivores globally [[7], [8], [9]]. The first human detection of RHEV was reported in Hong Kong in 2018, associated with persistent hepatitis following liver transplant [10]. Subsequent human infections caused by RHEV of genotype HEV-C1 have been reported in Canada, Spain, and France [[11], [12], [13]]. However, the precise route of RHEV transmission to humans remains unknown. These findings raise concerns regarding RHEV potentially being the causative agent for cases of unexplained hepatitis that might be underdiagnosed by current hepatitis E diagnostic tests.

In the European Union/European Economic Area (EU/EEA), the number of confirmed HEV cases increased tenfold between 2005 and 2015 [14], likely due to heightened awareness of HEV among European physicians. In the Västra Götaland Region (VGR) in western Sweden, where this study was conducted, only approximately 4–18 cases were reported annually during 2013–2023 [15], despite around 16% of blood donors in the region testing positive for anti-HEV IgG and 1% having detectable HEV RNA in serum [16]. Moreover, approximately 50 patients present annually with acute hepatitis of unknown etiology in VGR. Despite often being subclinical, the low number of identified cases in Sweden may suggest underdiagnosis of hepatitis E, possibly due to physicians' limited awareness of HEV or the presence of divergent HEV types undetectable by current diagnostic assays, such as the RHEV strains identified alongside human HEV-3 strains in wastewater from VGR [17]. These RHEV strains were genetically distinct from previously reported European and Asian strains. Whether divergent human HEV types or RHEV may underlie disease in patients diagnosed with hepatitis of unknown etiology remains uncertain.

Wastewater monitoring has proven effective in tracking the spread and circulation of enteric viruses within communities [18,19]. During the COVID-19 pandemic, this approach was widely employed to monitor SARS-CoV-2 globally. In our region, we developed the technology to, in addition to SARS-CoV-2, also monitor the spread of enteric viruses [20]. In this study, we utilized this developed technique to monitor the presence and concentration of HEV and RHEV in influent (incoming) wastewater in Gothenburg over one year. Our objectives included determining if the virus strains identified in wastewater are associated with those isolated from humans, wild boars, domestic pigs, and other types of water in the same area, as well as assessing the potential risk of human infections posed by RHEV in Sweden.

## 2 Materials and methods

### 2.1 Wastewater and lake water sampling

From the week 2 of 2023 until the week 52, influent wastewater samples were collected at the Rya wastewater treatment plant (WWTP) on a weekly basis. A total of 51 samples were acquired using a fixed-site sampler, collecting 30 mL per every 10,000 m^3^ influent wastewater. The daily flow-weighted samples were stored at 4 °C and pooled into a composite weekly sample every Monday. These samples were immediately sent to the Clinical Microbiology Laboratory (CML) at the Sahlgrenska University Hospital, Gothenburg for viral analysis. The Rya WWTP serves as the recipient of wastewater from the greater Gothenburg region, including storm water and snow-melting water from certain older sections of the city. The proportion of wastewater remains consistent throughout the year, whereas precipitation levels exhibit variations between weeks. The volume of each weekly sample ranged between 3.3 and 13.0 L throughout the year 2023. The comprehensive details regarding the sampling locations and procedures have been previously described [21].

In addition, water samples from five lakes proximate to human residences areas in the Gothenburg region were collected to assess the presence of HEV and RHEV. These 1-Liter grab lake samples were monthly collected from November 2023 to March 2024. During the winter months, several lakes were frozen and samples cannot be collected. Consequently, a total of 16 lake samples were collected and sent to CML at the Sahlgrenska University Hospital for analysis.

### 2.2 Concentration of viruses from influent wastewater

Upon the wastewater and lake samples arrival at CML, viruses were immediately concentrated using our in-house developed method. This method applies the utilization of NanoCeram electropositive filter (Argonide, Florida, USA) as the primary concentration method, followed by ultracentrifugation as secondary concentration method, which has been previously described [22]. After ultracentrifugation, the pellet was suspended in 2.4 mL Tris-HCl buffer (pH 8.0) and allowed to dissolve overnight. Subsequently, 1 mL of dissolved pellet was preserved for the extraction of viral nucleic acids, while the remaining was stored at −80 °C for further analysis.

### 2.3 Detection of HEV and RHEV by qPCR

The viral nucleic acids were extracted using the QIAamp Circulating Nucleic Acid Kit (Qiagen, Hilden, Germany) following the manufacturer's instructions. Two qPCRs were conducted to detect the presence of HEV and RHEV in wastewater and lake samples. For both qPCRs, the 20 μL reaction mixture contained 5 μL extracted nucleic acids, 4× UltraPlex 1-Step ToughMix (Quantabio, Beverly, USA), 0.5 μM of forward and reverse primer, 0.25 μM probe, and 7.5 μL nuclease-free water (Sigma-Aldrich, St. Louis, USA). The primers and probe sequences are provided in Supplementary Table 1. All qPCRs were performed with an initial reverse transcription cycle of 50 °C for 10 min and 95 °C for 10 min, followed by 45 cycles of 95 °C for 10 s and 60 °C for 1 min. The assays were performed on a QuantStudio 5 Real-Time PCR System (Applied Biosystems, Foster City, CA, USA), with each sample analyzed in duplicate. In each run, nuclease-free water was used as a negative control, and plasmids containing target regions of HEV and RHEV were synthesized by Eurofins Genomics (Ebersberg, Germany) and used as positive controls. Each plasmid was 10-fold serially diluted, and five dilutions were used to generate a standard curve. The concentration of viral genomes in wastewater was expressed as viral genome copies per mL by determining the mean Ct value of each sample and correlating it with concentrations from the standard curve.

### 2.4 Amplification of HEV and RHEV by semi/nested-PCR

Viral nucleic acids were reverse-transcribed into cDNA using the High-Capacity cDNA Reverse Transcription Kit (Applied Biosystems). The 20-μL reaction mix consisted of 10 μL extracted nucleic acids, 1× RT random primers, 1× RT buffer, 4 mM dNTPs, 1 μL RNase inhibitor, 1 μL MultiScribe Reverse Tanscriptase, and 3.2 μL nuclease-free water. The conditions for cDNA synthesis were set at 25 °C for 10 min, 37 °C for 120 min, and a final step at 85 °C for 5 min. Afterwards, two PCR systems, broadly targeting both HEV and RHEV, where one nested PCR targeting the RNA-dependent RNA polymerase (RdRp) region [23] and one in-house developed semi-nested PCR targeting junction region of ORF1-ORF2-ORF3, were applied to amplify genetic materials from both human and rodent sources in influent wastewater samples. The PCR reaction mixtures for both systems were identical, except for the primers. For the first round amplification, the 50-μL reaction mix contained 5 μL cDNA, 1× Taq buffer (Applied Biosystems), 2.25 mM MgCl_2_ (Applied Biosystems), 0.2 mM dNTPs (Sigma-Aldrich), 1 U Taq DNA polymerase (Roche Diagnostics), 0.3 μM of each primer, and 3.2 μL nuclease-free water. PCR amplification involved an initial cycle at 94 °C for 3 min, followed by 40 cycles at 94 °C for 20 s, annealing at 50 °C or 58 °C for 30 s, extension at 72 °C for 1 min, and a final extension at 72 °C for 5 min. For the nested PCRs, 5 μL of the product from the first round of amplification was used, with the same reaction mix and cycling conditions, except for the primers. All primer information is provided in Supplementary Table 1. Each sample was analyzed in duplicate. PCR products were visualized on a 1.5% agarose gel electrophoresis, and those with clear band at correct size were purified using the QIAquick PCR purification kit (Qiagen) and sent to Eurofins Genomics (Germany) for Sanger sequencing.

### 2.5 Phylogenetic analysis

Sequence quality was manually assessed before assembly into contigs using BioEdit software (Version 7.2.5). HEV sequences, amplified from the RdRp region (339 bp in length), were aligned with corresponding regions from 74 HEV-3 sequences and 5 reference sequences from other HEV genotypes. Additionally, twelve sequences isolated from patients between late 2022 and early 2024 were included in the analysis. As the reference laboratory for HEV in Sweden, we receive suspected HEV-infected samples not only from Gothenburg but also from other regions across the country for confirmatory HEV diagnosis. Of these twelve samples, three were collected from the Gothenburg region, while the remaining nine were from Stockholm region (*n* = 3), Skåne region (*n* = 2), Gävleborg region (*n* = 2), Halland region (*n* = 1), and Jämtland Härjedalen region (*n* = 1). The HEV sequences, amplified from the junction region (461 bp in length), were aligned with 52 HEV-3 sequences and 3 reference sequences from other HEV genotypes. RHEV sequences, amplified from the RdRp region (378 bp in length), were aligned with 55 RHEV sequences along with one HEV-1 and one HEV-3 sequence as the outgroup. All reference sequences were retrieved from the GenBank database. Alignments were performed using MAFFT software (Version 7.520). The evolutionary distances were estimated using DNADIST program in the PHYLIP package (Version 3.698). Phylogenetic trees were constructed using the unweight pair-group method using arithmetic averages (UPGMA) method in MEGA11 software. All sequences obtained in this study have been deposited in GenBank under the accession numbers PP526293-PP526315 and PP532697-PP532708.

## 3 Results

### 3.1 Prevalence and quantification of HEV and RHEV in influent wastewater by qPCR

**Fig. 1:**
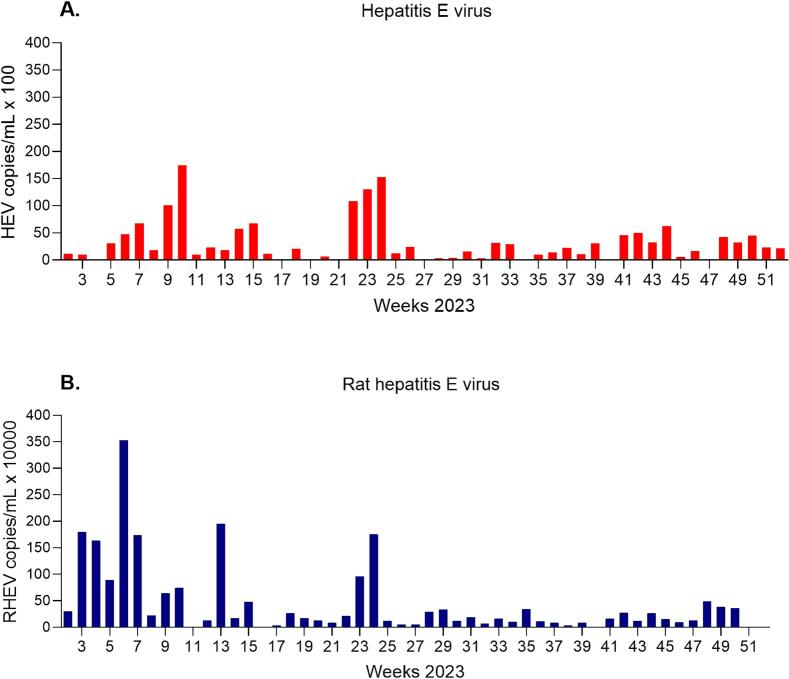
The concentration of HEV (A) and RHEV (B) in weekly wastewater during 2023.

HEV RNA could be detected in 41 of 51 (86%) weekly wastewater samples during 2023 by qPCR (Table 1). The RNA concentration ranged from 180 to 17,500 viral copies per mL wastewater. Two peaks of HEV genome were observed, one occurred in February/March during weeks 9–10 and the second in May/June during weeks 22–24 (Fig. 1A). In comparison, both the prevalence and concentration of RHEV exceeded those of HEV. RHEV was present in 50 of 51 (98%) wastewater samples, with concentrations ranging from 2.86 × 10^3^ to 3.53 × 10^6^ viral copies per mL (Fig. 1B). The seasonal trend for RHEV was similar to that for HEV, with elevated viral RNA concentrations observed in the first half of 2023, peaking at week 6 (Fig. 1B). Furthermore, neither HEV nor RHEV were detected in 16 samples collected from five lakes within the same region by qPCR.

**Table 1:** The concentration (genome copies per mL), sequencing and genotyping of HEV and RHEV in weekly wastewater samples during 2023.

Sample	Week (2023)	HEV		RHEV	
		qPCR (Genome copies/mL)	Sequencing (subtype)	qPCR (Genome copies/mL)	Sequencing
WP23–1	2	1.12E+03	Negative	3.05E+05	Negative
WP23–2	3	1.02E+03	Negative	1.80E+06	Positive
WP23–3	4	ND⁎	Negative	1.63E+06	Negative
WP23–4	5	3.05E+03	Negative	8.91E+05	Positive
WP23–5	6	4.79E+03	Negative	3.53E+06	Positive
WP23–6	7	6.74E+03	Positive (3c/i)	1.74E+06	Negative
WP23–7	8	1.83E+03	Negative	2.21E+05	Negative
WP23–8	9	1.01E+04	Positive (3c/i)	6.49E+05	Negative
WP23–9	10	1.75E+04	Positive (3c/i)	7.49E+05	Positive
WP23–10	11	9.91E+02	Negative	ND	Positive
WP23–11	12	2.33E+03	Negative	1.37E+05	Negative
WP23–12	13	1.77E+03	Positive (3b)	1.95E+06	Positive
WP23–13	14	5.73E+03	Positive (3c/i)	1.75E+05	Negative
WP23–14	15	6.70E+03	Negative	4.86E+05	Negative
WP23–15	16	1.18E+03	Negative	2.86E+03	Negative
WP23–16	17	ND	Negative	3.78E+04	Negative
WP23–17	18	2.06E+03	Positive (unclassified)	2.62E+05	Negative
WP23–18	19	ND	Positive (3c/i)	1.70E+05	Negative
WP23–19	20	6.65E+02	Negative	1.38E+05	Positive
WP23–20	21	ND	Negative	8.39E+04	Positive
WP23–21	22	1.09E+04	Positive (3c/i)	2.17E+05	Negative
WP23–22	23	1.30E+04	Positive (unclassified)	9.69E+05	Negative
WP23–23	24	1.52E+04	Negative	1.76E+06	Positive
WP23–24	25	1.21E+03	Negative	1.25E+05	Negative
WP23–25	26	2.42E+03	Negative	5.22E+04	Negative
WP23–26	27	ND	Negative	5.22E+04	Negative
WP23–27	28	3.30E+02	Negative	2.99E+05	Positive
WP23–28	29	4.24E+02	Negative	3.39E+05	Positive
WP23–29	30	1.58E+03	Negative	1.26E+05	Positive
WP23–30	31	2.79E+02	Negative	1.91E+05	Positive
WP23–31	32	3.16E+03	Positive (3c/i)	7.63E+04	Negative
WP23–32	33	2.91E+03	Negative	1.60E+05	Positive
WP23–33	34	ND	Negative	1.03E+05	Negative
WP23–34	35	9.55E+02	Positive (unclassified)	3.47E+05	Negative
WP23–35	36	1.42E+03	Positive (3c/i)	1.15E+05	Negative
WP23–36	37	2.26E+03	Negative	8.80E+04	Positive
WP23–37	38	1.07E+03	Negative	3.88E+04	Positive
WP23–38	39	3.05E+03	Negative	8.74E+04	Negative
WP23–39	40	1.81E+02	Negative	8.80E+03	Positive
WP23–40	41	4.58E+03	Negative	1.70E+05	Negative
WP23–41	42	4.97E+03	Positive (3c/i)	2.70E+05	Negative
WP23–42	43	3.23E+03	Negative	1.25E+05	Negative
WP23–43	44	6.23E+03	Negative	2.62E+05	Negative
WP23–44	45	5.90E+02	Negative	1.56E+05	Positive
WP23–45	46	1.68E+03	Negative	9.94E+04	Negative
WP23–46	47	ND	Negative	1.30E+05	Negative
WP23–47	48	4.22E+03	Positive (3c/i)	4.99E+05	Negative
WP23–48	49	3.24E+03	Negative	3.85E+05	Negative
WP23–49	50	4.51E+03	Negative	3.61E+05	Positive
WP23–50	51	2.32E+03	Negative	5.12E+03	Positive
WP23–51	52	2.17E+03	Negative	3.26E+03	Negative

⁎ ND = Not determined.

### 3.2 Phylogenetic analysis of HEV and RHEV in wastewater

Two broad-target PCRs were employed to amplify HEV and RHEV sequences from wastewater samples. Bands of the correct size were visualized in 36 out of 51 wastewater samples targeting the RdRp region, from which sequences were successfully assembled in 33 samples. BLAST analysis showed that 12 of these sequences belonged to HEV, while 21 sequences were identified as RHEV. Additionally, five samples yielded correct bands using the PCR system targeting the junction region. However, only two sequences were successfully assembled due to either low-quality reads or the presence of multiple peaks in the remaining three sequences. In these two sequences, parts of the 3′-end were missing, and only the segments corresponding to the end of ORF1 (positions 4560–5022; NC-001434) were successfully assembled. Both sequences were identified as HEV.

**Fig. 2:**
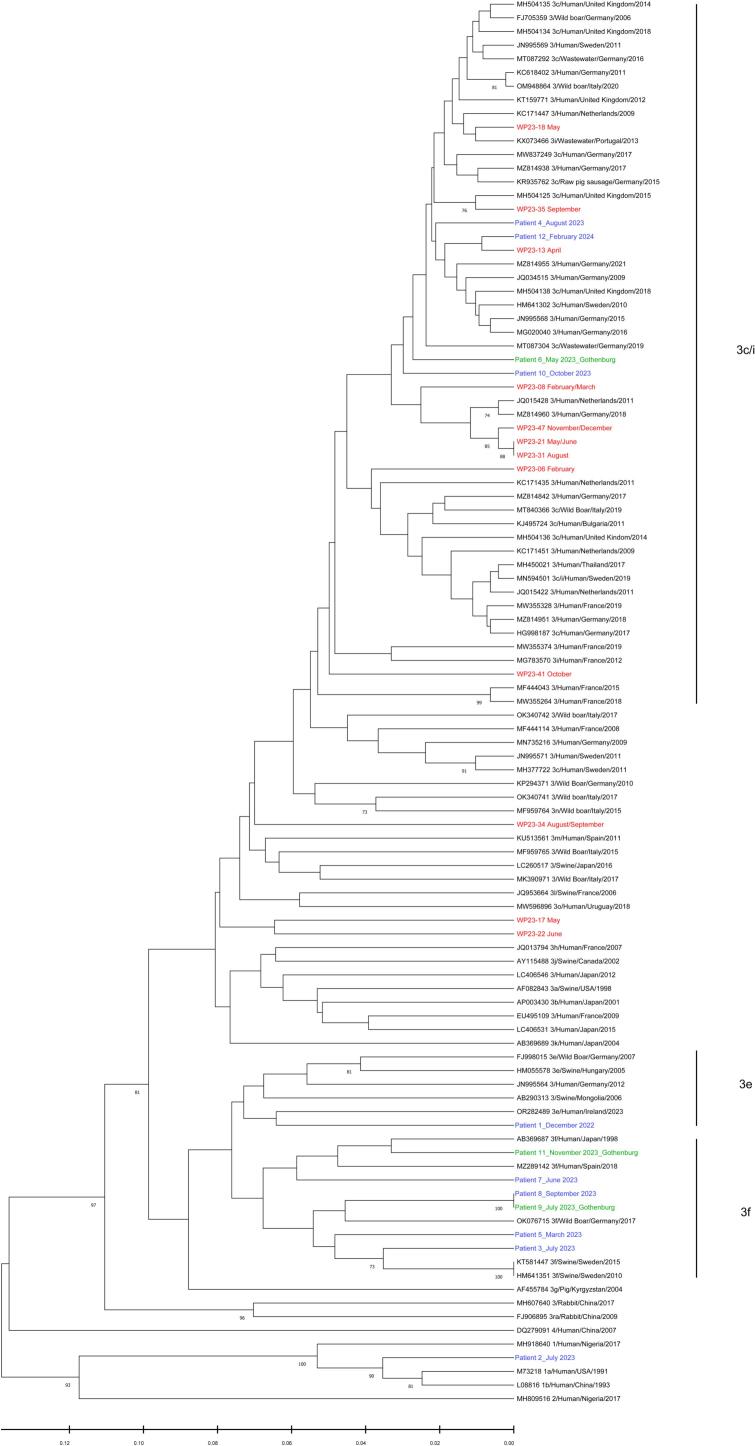
Phylogenetic tree of HEV RdRp region. Phylogenetic tree was constructed based on the 339 nucleotides of partial HEV RdRp region using UPGMA method. Red labels represent sequences detected in wastewater samples collected during 2023. Green labels represent sequences isolated from clinical samples within the Gothenburg region during 2023. Blue labels represent sequences isolated from clinical samples from other regions in Sweden. Sequences belonging to subtype 3c/i, 3f, and 3e are highlighted on the right of the phylogenetic tree. The numbers on the tree branches represent bootstrap values, with values greater than 70 shown. (For interpretation of the references to colour in this figure legend, the reader is referred to the web version of this article.)

**Supplementary Fig. 1:**
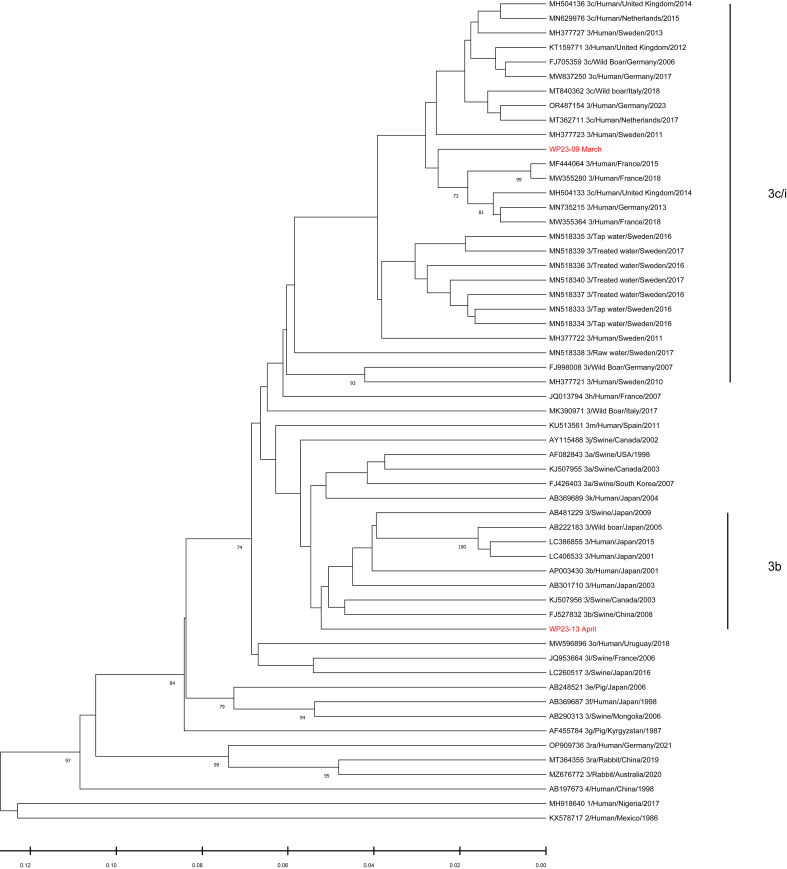
Phylogenetic tree of HEV ORF1-ORF2-ORF3 junction region. Only the segments corresponding to the end of ORF1 (positions 4560-5022; NC-001434) were successfully assembled and used for the phylogenetic analysis. Red labels represent sequences detected in wastewater samples collected during 2023 from this study. Sequences belonging to subtype 3c/i and 3b are highlighted on the right of the phylogenetic tree. The numbers on the tree branches represent bootstrap values, with values greater than 70 shown.

Phylogenetic analysis of the RdRp region revealed that all HEV sequences obtained from wastewater were HEV-3. The majority of these sequences (9 out of 12) were classified within subtype 3c/i, while the remaining three sequences (WP23–17/22/34) could not be assigned to any existing subtypes (Fig. 2). HEV sequences isolated from patients had a different pattern. Most sequences (6 out of 12) were classified as subtype 3f, showing genetic similarities to strains identified in swine and wild boars in Sweden and other European countries (Fig. 2). Additionally, four sequences (Patient 4/6/10/12) grouped within the subtype 3c/i, one of them (Patient 12) closely related to strains identified in wastewater. Another patient strain (Patient 1) was identified as subtype 3e, a subtype commonly found in domestic pigs in Sweden. The last sequence from patients belonged to HEV-1, which could be a possible travel-associated infection. Phylogenetic analysis of the junction region revealed that one strain from wastewater was subtype 3c/i. The other one was subtype 3b, which is commonly found in China and Japan, but not previously reported in Sweden (Supplementary Fig. 1).

**Fig. 3:**
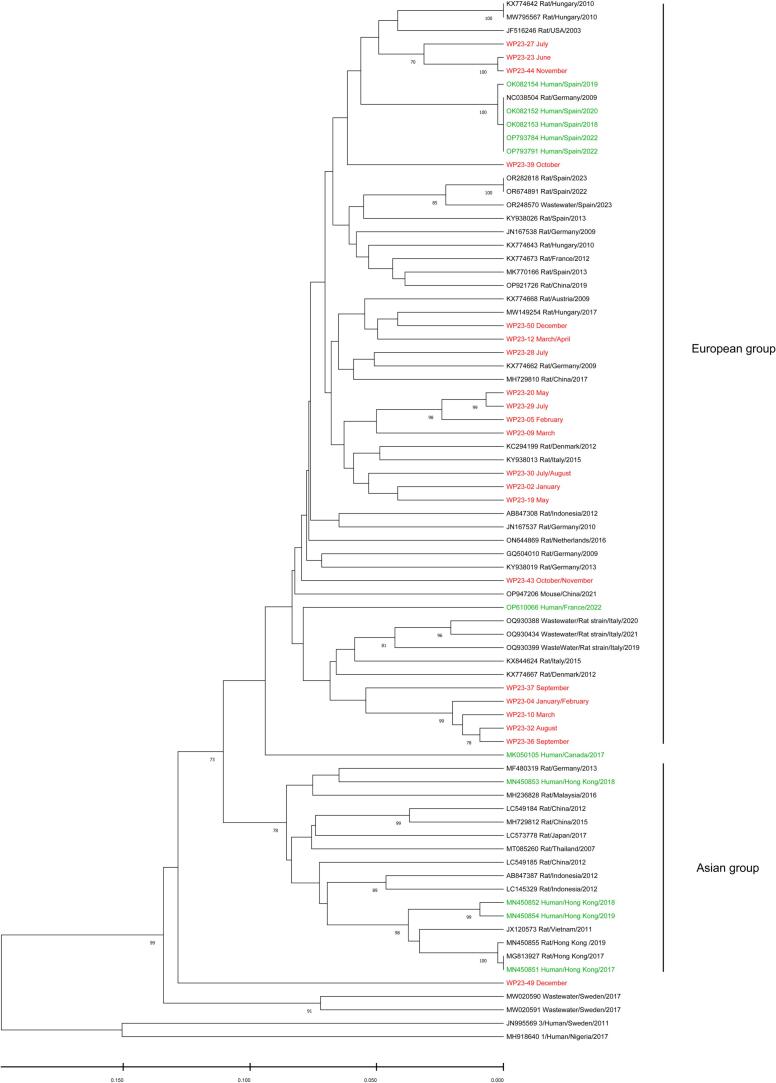
Phylogenetic tree of RHEV RdRp region. Phylogenetic tree was constructed based on the 378 nucleotides of partial RHEV RdRp region using UPGMA method. Red labels represent sequences detected in wastewater samples collected during 2023 from this study. Green labels represent sequences previously reported associated with human infections. HEV genotypes 1 and 3 were used as outgroup strains. The RHEV sequences are grouped into two major clusters, tentatively named the Asian and European groups. The numbers on the tree branches represent bootstrap values, with values greater than 70 shown. (For interpretation of the references to colour in this figure legend, the reader is referred to the web version of this article.)

Phylogenetic analysis of the RHEV RdRp region revealed two major groups for RHEV strains, one group with RHEV strains mainly isolated from Europe, and another from Asia (Fig. 3). Nearly all of our RHEV sequences (20 out of 21) identified in wastewater fell into the European group. Most of these strains (18 out of 20) could be further categorized into four distinct clades, clustering closely with RHEV strains previously identified in rodents across other European countries. Notably, one sequence (WP23–49) had significant genetic divergence from all known RHEV strains, placing it outside both the European and Asian groups. Furthermore, all RHEV sequences obtained in this study differed from those previously identified in human cases.

## 4 Discussion

Our year-long surveillance in Gothenburg, Sweden, revealed a notable prevalence of HEV genomes in influent wastewater. This observed prevalence exceeds that reported from other European countries, where HEV-3 is also the dominant genotype [[24], [25], [26], [27]]. However, despite the high prevalence, the concentration of HEV genomes remained relatively low compared to SARS-CoV-2 and other common human enteric viruses, such as norovirus GII, astrovirus, rotavirus, and adenovirus, which were concurrently monitored in the same region during the pandemic [20,28]. Despite being a notifiable infectious disease in Sweden, only seven cases were reported in the VGR region during 2023 [15], alongside approximately 16% of blood donors testing positive for anti-HEV IgG and 1% having detectable HEV RNA in serum within the same region [16]. Our wastewater surveillance implies a significant underestimation of hepatitis E infections in the region and potentially throughout Sweden. This discrepancy may stem from asymptomatic individuals not seeking medical help or physicians lacking awareness of the infection. In addition, the constant emergence of new HEV subtypes, genotypes, or even more genetically divergent variants could potentially go undetected by current clinical diagnostic methods. These findings underscore the importance of raising awareness regarding HEV transmission in the community and the urgent need for a thorough evaluation of clinical diagnosis methods for HEV to address the challenges posed by this virus.

Phylogenetic analysis revealed a predominance of subtype 3c/i among HEV sequences from wastewater, contrasting with the prevalence of subtype 3f observed in patients in 2023. Our study employed nested-PCR followed by Sanger sequencing, which may only detect the dominant strain in the event of co-circulation with more than one subtype, potentially missing subtype 3f when its concentration is significantly lower than that of 3c/i. Several studies have reported that infections with subtype 3f were associated with higher virus load and more frequent hospitalization compared to those infected with subtype 3c [29,30]. Conversely, our previous work examining HEV strains isolated from liver transplant recipients, who are at increased risk of chronic infections, found that all sequences belonged to the subtype 3c/i [31], aligning with findings from other European studies where subtype 3c/i is more prevalent in chronic hepatitis E patients [32,33]. The possibility that subtype 3c/i may result in prolonged viral shedding, especially in individuals with chronic infections, could explain the higher detection rate in wastewater. However, whether differences in virulence, viral loads, and shedding periods between subtypes 3f and 3c/i account for the observed discrepancy between wastewater and clinical samples remains unclear. Despite these considerations, the consistent absence of subtype 3f in wastewater throughout the year is notable. In addition, our previous studies found that the dominant subtype in Swedish wild boar and domestic pigs are 3f, while the 3c/i in raw water for drinking water treatment plant and in tap water [6,34,35]. It is suspected that HEV-3 transmission in Sweden is not limited to zoonotic routes but may also occur through other pathways, such as waterborne transmission. Future studies should focus on comparing viral loads and shedding durations in feces from individuals infected with different HEV-3 subtypes and potential new transmission routes for HEV3 to better understand the reasons behind the distinct patterns observed in wastewater and clinical samples.

We also identified sequences belonging to subtype 3b in wastewater, previously unreported in Sweden until March 2024 when this subtype was first detected in a clinical patient sample through routine testing. This underscores the value of wastewater monitoring as a complementary approach to clinical surveillance for identifying new HEV subtypes within the community, as recently demonstrated in Italy and Germany [36,37]. Regular monitoring of HEV in wastewater on a weekly or monthly basis will enhance our understanding of the emergence of new genotypes or subtypes and potential shifts in genetic dynamics. In addition, three sequences could not be assigned to any existing subtypes in our phylogenetic analysis. This may be due to the presence of new or unclassified subtypes, or the short genomic length of the sequences. Obtaining complete or longer HEV genomes may provide more comprehensive information for accurate typing. However, amplifying complete HEV genomes from wastewater using traditional Sanger sequencing presents challenges due to the time and resource demands, as well as the complex composition of wastewater. In this context, new methods, such as Nanopore sequencing technology, could facilitate the acquisition of longer reads from wastewater samples.

In our previous study, we identified RHEV in effluent (outgoing) wastewater in the Rya WWTP through Next Generation Sequencing [17]. In this investigation, we comprehensively monitored RHEV in influent wastewater, revealing its consistent detection in nearly all weekly samples. This prevalence aligns with recent findings in Spain, reporting a 94% prevalence of RHEV in wastewater [38]. Similarly, monitoring in Italy showed RHEV RNA detection in 44% of all wastewater samples using a broad-spectrum PCR approach [39]. Furthermore, quantification of RHEV genomes in our study indicated concentrations approximately 1–4 log10 higher than those of HEV genomes. These results suggest RHEV is prevalent in wastewater across European countries, likely attributed to rodents harboring the virus and residing in wastewater pipeline systems. In addition, some rodent species, such as guinea pigs, hamsters, and rats, are occasionally kept as household pets in this region. Various rodent species have been shown to carry both HEV and RHEV [40]. Moreover, common household companion pets, including dogs and cats, have also been identified as potential carriers of these viruses [9,41]. These animals can excrete the viruses in their feces, contributing to the presence of HEV and RHEV in influent wastewater. The extent to which HEV and/or RHEV are present in domestic pets, and their potential role as alternative zoonotic sources of virus transmission, remains unclear and warrants further investigation. Given the potential for interaction between rodents, pets, and humans, especially in urban environments, we investigated the presence of HEV and RHEV in lakes in proximity to human residences but found no trace. However, considering the potentially low concentration of both viruses in environmental waters, the 1-L grab samples from these lakes may have been insufficient to detect the viruses. Therefore, the absence of RHEV in these lake samples does not diminish its increasing significance as a public health concern. Analyzing larger volumes of environmental samples and including a broader spectrum of water sources, particularly those near pig farms and abattoirs where rats harboring RHEV have been captured previously [42], could better clarify the transmission routes of RHEV and assess its potential risk to public health.

We observed a high degree of genetic diversity among RHEV strains in wastewater during the one-year monitoring, with strains distributed across different branches of the phylogenetic tree. This finding contrasts with recent research in Italy, which reported genetic similarity among RHEV strains from the same WWTP, indicating a clear geographic or WWTP-related pattern [39]. Our results suggest the possibility of multiple sources or multiple animal hosts contributing to this diversity. Moreover, one RHEV strain was notably distinct from previously identified strains in humans, rodents, and environmental samples, potentially indicating the emergence of a genetically divergent RHEV lineage or clade. The transmission route from RHEV to humans remains unknown [43]. In Sweden, a proportion of patients exhibit hepatitis symptoms without an identified source of infection. Whether RHEV is responsible for these cases remains unclear, as current routine clinical diagnostic methods do not detect RHEV. Although none of the strains identified in this study were genetically similar to those associated with human infections in Hong Kong, Canada, France, and Spain, the risk should not be ignored. The widespread presence and high diversity of RHEV in wastewater underscore its circulation among animal hosts, suggesting potential zoonotic transmission to humans. Therefore, continuous investigation is essential to determine whether RHEV contributes to cases of hepatitis with unknown etiology in the region.

In conclusion, this study reveals the prevalence and genetic diversity of HEV and RHEV in wastewater in Gothenburg. The notable discrepancy between the prevalence and identified subtypes of HEV-3 in wastewater and clinical samples suggests that many HEV infections remain undetected. There may also be differences in viral loads and shedding durations in human feces between HEV-3 subtypes. These findings underscore the need for improved clinical diagnostics and enhanced awareness of HEV transmission patterns. Moreover, the consistent detection and high diversity of RHEV in wastewater underscore the importance of comprehensive environmental surveillance to understand the transmission routes of RHEV. Future efforts should focus on exploring the potential role of RHEV in hepatitis cases of unknown etiology, as the current diagnostic kits available in our clinical laboratory do not include RHEV, potentially leading to its undetected presence even when testing for HEV.

The following are the supplementary data related to this article.

Supplementary Table 1 Primers and probes used for the detection and amplification of HEV and RHEV. Supplementary Table 1

## CRediT authorship contribution statement

**Marianela Patzi Churqui:** Writing – review & editing, Visualization, Software, Methodology, Investigation. **Margarita Ghaleb:** Validation, Software, Investigation, Formal analysis, Data curation. **Timur Tunovic:** Methodology, Investigation. **Miriam Frankal:** Resources. **Lucica Enache:** Resources. **Kristina Nyström:** Writing – review & editing, Resources. **Martin Lagging:** Writing – review & editing, Supervision, Resources. **Hao Wang:** Writing – review & editing, Writing – original draft, Supervision, Methodology, Investigation, Funding acquisition, Formal analysis, Data curation, Conceptualization.

## Declaration of competing interest

The authors declare that they have no known competing financial interests or personal relationships that could have appeared to influence the work reported in this article.

## Data Availability

Data will be made available on request.

## References

[1] Purdy M.A., Drexler J.F., Meng X.J., Norder H., Okamoto H., Van der Poel W.H.M., Reuter G., de Souza W.M., Ulrich R.G., Smith D.B. (2022). ICTV virus taxonomy profile: Hepeviridae 2022. J. Gen. Virol..

[2] (2022). International Committee on Taxonomy of Viruses Hepeviridae Study, Family: Hepeviridae. https://ictv.global/report/chapter/hepeviridae/hepeviridae.

[3] Smith D.B., Izopet J., Nicot F., Simmonds P., Jameel S., Meng X.J., Norder H., Okamoto H., van der Poel W.H.M., Reuter G., Purdy M.A. (2020). Update: proposed reference sequences for subtypes of hepatitis E virus (species Orthohepevirus A). J. Gen. Virol..

[4] Yugo D.M., Meng X.J. (2013). Hepatitis E virus: foodborne, waterborne and zoonotic transmission. Int. J. Environ. Res. Public Health.

[5] Denner J., Pischke S., Steinmann E., Blumel J., Glebe D. (2019). Why all blood donations should be tested for hepatitis E virus (HEV). BMC Infect. Dis..

[6] Wang H., Kjellberg I., Sikora P., Rydberg H., Lindh M., Bergstedt O., Norder H. (2020). Hepatitis E virus genotype 3 strains and a plethora of other viruses detected in raw and still in tap water. Water Res..

[7] Johne R., Heckel G., Plenge-Bonig A., Kindler E., Maresch C., Reetz J., Schielke A., Ulrich R.G. (2010). Novel hepatitis E virus genotype in Norway rats, Germany. Emerg. Infect. Dis..

[8] Bai H., Li W., Guan D., Su J., Ke C., Ami Y., Suzaki Y., Takeda N., Muramatsu M., Li T.C. (2020). Characterization of a novel rat hepatitis E virus isolated from an Asian musk shrew (*Suncus murinus*). Viruses.

[9] Caballero-Gomez J., Rivero-Juarez A., Jurado-Tarifa E., Jimenez-Martin D., Jimenez-Ruiz E., Castro-Scholten S., Ulrich R.G., Lopez-Lopez P., Rivero A., Garcia-Bocanegra I. (2022). Serological and molecular survey of hepatitis E virus in cats and dogs in Spain. Transbound. Emerg. Dis..

[10] Sridhar S., Yip C.C.Y., Wu S., Cai J., Zhang A.J., Leung K.H., Chung T.W.H., Chan J.F.W., Chan W.M., Teng J.L.L., Au-Yeung R.K.H., Cheng V.C.C., Chen H., Lau S.K.P., Woo P.C.Y., Xia N.S., Lo C.M., Yuen K.Y. (2018). Rat hepatitis E virus as cause of persistent hepatitis after liver transplant. Emerg. Infect. Dis..

[11] Andonov A., Robbins M., Borlang J., Cao J., Hatchette T., Stueck A., Deschambault Y., Murnaghan K., Varga J., Johnston L. (2019). Rat hepatitis E virus linked to severe acute hepatitis in an immunocompetent patient. J. Infect. Dis..

[12] Rivero-Juarez A., Frias M., Perez A.B., Pineda J.A., Reina G., Fuentes-Lopez A., Freyre-Carrillo C., Ramirez-Arellano E., Alados J.C., Rivero A., Hepavir, G.-S. Groups (2022). Orthohepevirus C infection as an emerging cause of acute hepatitis in Spain: first report in Europe. J. Hepatol..

[13] Rodriguez C., Marchand S., Sessa A., Cappy P., Pawlotsky J.M. (2023). Orthohepevirus C hepatitis, an underdiagnosed disease?. J. Hepatol..

[14] ECDC Hepatitis E in the EU/EEA, 2005–2015. https://www.ecdc.europa.eu/en/publications-data/hepatitis-e-eueea-2005-2015.

[15] (2024). Folkhälsomyndigheten, Hepatit E - sjukdomsstatistik. https://www.folkhalsomyndigheten.se/folkhalsorapportering-statistik/statistik-a-o/sjukdomsstatistik/hepatit-e/?t=county.

[16] Norder H., Karlsson M., Mellgren A., Konar J., Sandberg E., Lasson A., Castedal M., Magnius L., Lagging M. (2016). Diagnostic performance of five assays for anti-hepatitis E virus IgG and IgM in a large cohort study. J. Clin. Microbiol..

[17] Wang H., Neyvaldt J., Enache L., Sikora P., Mattsson A., Johansson A., Lindh M., Bergstedt O., Norder H. (2020). Variations among viruses in influent water and effluent water at a wastewater plant over one year as assessed by quantitative PCR and metagenomics. Appl. Environ. Microbiol..

[18] Ndiaye A.K., Diop P.A., Diop O.M. (2014). Environmental surveillance of poliovirus and non-polio enterovirus in urban sewage in Dakar, Senegal (2007-2013). Pan Afr. Med. J..

[19] Hellmer M., Paxeus N., Magnius L., Enache L., Arnholm B., Johansson A., Bergstrom T., Norder H. (2014). Detection of pathogenic viruses in sewage provided early warnings of hepatitis A virus and norovirus outbreaks. Appl. Environ. Microbiol..

[20] Wang H., Churqui M.P., Tunovic T., Enache L., Johansson A., Lindh M., Lagging M., Nystrom K., Norder H. (2023). Measures against COVID-19 affected the spread of human enteric viruses in a Swedish community, as found when monitoring wastewater. Sci. Total Environ..

[21] Saguti F., Magnil E., Enache L., Churqui M.P., Johansson A., Lumley D., Davidsson F., Dotevall L., Mattsson A., Trybala E., Lagging M., Lindh M., Gisslen M., Brezicka T., Nystrom K., Norder H. (2021). Surveillance of wastewater revealed peaks of SARS-CoV-2 preceding those of hospitalized patients with COVID-19. Water Res..

[22] Wang H., Sikora P., Rutgersson C., Lindh M., Brodin T., Bjorlenius B., Larsson D.G.J., Norder H. (2018). Differential removal of human pathogenic viruses from sewage by conventional and ozone treatments. Int. J. Hyg. Environ. Health.

[23] Johne R., Plenge-Bonig A., Hess M., Ulrich R.G., Reetz J., Schielke A. (2010). Detection of a novel hepatitis E-like virus in faeces of wild rats using a nested broad-spectrum RT-PCR. J. Gen. Virol..

[24] Takuissu G.R., Kenmoe S., Ndip L., Ebogo-Belobo J.T., Kengne-Nde C., Mbaga D.S., Bowo-Ngandji A., Oyono M.G., Kenfack-Momo R., Tchatchouang S., Kenfack-Zanguim J., Lontuo Fogang R., Zeuko'o Menkem E., Kame-Ngasse G.I., Magoudjou-Pekam J.N., Nkie Esemu S., Veneri C., Mancini P., Bonanno Ferraro G., Iaconelli M., Suffredini E., La Rosa G. (2022). Hepatitis E virus in water environments: a systematic review and meta-analysis. Food Environ. Virol..

[25] Di Profio F., Melegari I., Palombieri A., Sarchese V., Arbuatti A., Fruci P., Marsilio F., Martella V., Di Martino B. (2019). High prevalence of hepatitis E virus in raw sewage in Southern Italy. Virus Res..

[26] Masclaux F.G., Hotz P., Friedli D., Savova-Bianchi D., Oppliger A. (2013). High occurrence of hepatitis E virus in samples from wastewater treatment plants in Switzerland and comparison with other enteric viruses. Water Res..

[27] Beyer S., Szewzyk R., Gnirss R., Johne R., Selinka H.C. (2020). Detection and characterization of hepatitis E virus genotype 3 in wastewater and urban surface waters in Germany. Food Environ. Virol..

[28] Wang H., Churqui M.P., Tunovic T., Enache L., Johansson A., Karmander A., Nilsson S., Lagging M., Andersson M., Dotevall L., Brezicka T., Nystrom K., Norder H. (2022). The amount of SARS-CoV-2 RNA in wastewater relates to the development of the pandemic and its burden on the health system. iScience.

[29] Subissi L., Peeters M., Lamoral S., Klamer S., Suin V., Van Gucht S. (2019). Subtype-specific differences in the risk of hospitalisation among patients infected with hepatitis E virus genotype 3 in Belgium, 2010-2018. Epidemiol. Infect..

[30] Abravanel F., Dimeglio C., Castanier M., Péron J.M., Kamar N., Lhomme S., Izopet J. (2020). Does HEV-3 subtype play a role in the severity of acute hepatitis E?. Liver Int..

[31] Frankal M., Skoglund C., Castedal M., Lagging M., Norder H. (2022). Hepatitis E virus infection, a risk for liver transplant recipients in Sweden. Transplant. Direct.

[32] Wang B., Harms D., Papp C.P., Niendorf S., Jacobsen S., Lutgehetmann M., Pischke S., Wedermeyer H., Hofmann J., Bock C.T. (2018). Comprehensive molecular approach for characterization of hepatitis E virus genotype 3 variants. J. Clin. Microbiol..

[33] Moal V., Gerolami R., Ferretti A., Purgus R., Devichi P., Burtey S., Colson P. (2014). Hepatitis E virus of subtype 3i in chronically infected kidney transplant recipients in southeastern France. J. Clin. Microbiol..

[34] Wang H., Karlsson M., Lindberg M., Nystrom K., Norder H. (2019). Hepatitis E virus strains infecting Swedish domestic pigs are unique for each pig farm and remain in the farm for at least 2 years. Transbound. Emerg. Dis..

[35] Wang H., Castillo-Contreras R., Saguti F., Lopez-Olvera J.R., Karlsson M., Mentaberre G., Lindh M., Serra-Cobo J., Norder H. (2019). Genetically similar hepatitis E virus strains infect both humans and wild boars in the Barcelona area, Spain, and Sweden. Transbound. Emerg. Dis..

[36] Rau F., Elsner C., Meister T.L., Gomer A., Kallies R., Dittmer U., Steinmann E., Todt D. (2024). Monitoring of hepatitis E virus in wastewater can identify clinically relevant variants, liver international: official journal of the International Association for the Study of the. Liver.

[37] Veneri C., Brandtner D., Mancini P., Bonanno Ferraro G., Iaconelli M., Del Giudice C., Ciccaglione A.R., Bruni R., Equestre M., Marcantonio C., Suffredini E., La Rosa G. (2024). Detection and full genomic sequencing of rare hepatitis E virus genotype 4d in Italian wastewater, undetected by clinical surveillance. Sci. Total Environ..

[38] Casares-Jimenez M., Garcia-Garcia T., Suarez-Cardenas J.M., Perez-Jimenez A.B., Martin M.A., Caballero-Gomez J., Michan C., Corona-Mata D., Risalde M.A., Perez-Valero I., Guerra R., Garcia-Bocanegra I., Rivero A., Rivero-Juarez A., Garrido J.J. (2024). Correlation of hepatitis E and rat hepatitis E viruses urban wastewater monitoring and clinical cases. Sci. Total Environ..

[39] Palombieri A., Di Profio F., Sarchese V., Fruci P., Suffredini E., Martella V., Veneri C., Bonanno Ferraro G., Mancini P., La Rosa G., Di Martino B. (2023). Surveillance for rat hepatitis E in wastewater networks, Italy. Microbiol. Spect..

[40] Porea D., Raileanu C., Crivei L.A., Gotu V., Savuta G., Pavio N. (2023). First detection of hepatitis E virus (Rocahepevirus ratti genotype C1) in Synanthropic Norway rats (*Rattus norvegicus*) in Romania. Viruses.

[41] Shun E.H., Situ J., Tsoi J.Y., Wu S., Cai J., Lo K.H., Chew N.F., Li Z., Poon R.W., Teng J.L., Cheng V.C., Yuen K.Y., Sridhar S. (2024). Rat hepatitis E virus (*Rocahepevirus ratti*) exposure in cats and dogs, Hong Kong. Emerg. Microbes Infect..

[42] De Sabato L., Ianiro G., Monini M., De Lucia A., Ostanello F., Di Bartolo I. (2020). Detection of hepatitis E virus RNA in rats caught in pig farms from northern Italy. Zoonoses Public Health.

[43] Sridhar S., Yip C.C., Wu S., Chew N.F., Leung K.H., Chan J.F., Zhao P.S., Chan W.M., Poon R.W., Tsoi H.W., Cai J.P., Chan H.S., Leung A.W., Tse C.W., Zee J.S., Tsang O.T., Cheng V.C., Lau S.K., Woo P.C., Tsang D.N., Yuen K.Y. (2021). Transmission of rat hepatitis E virus infection to humans in Hong Kong: a clinical and epidemiological analysis. Hepatology.

