# Supplementary material for: High prevalence of hepatitis E and rat hepatitis E viruses in wastewater in Gothenburg, Sweden
Source: One Health. 2024 Aug 22;19:100882. doi: 10.1016/j.onehlt.2024.100882 (PMC11391864; doi:10.1016/j.onehlt.2024.100882)
Supplement: Supplementary Table 1 — Primers and probes used for the detection and amplification of HEV and RHEV. [file mmc2.docx]

Supplementary Table 1. Primers and probes used for the detection and amplification of HEV and RHEV.

| Target | Primer/Probe | Primer and Probe sequence |
| --- | --- | --- |
| RHEV (qPCR) | Rat-HEV F | TACTGCTAGAGAGGCCCAG |
|  | Rat-HEV R | GCTGTATCGGATGCGACC |
|  | Rat-HEV-P | FAM-ACCGCCTTTGCTAATGCT-MGB |
| HEV (qPCR) | JVHEV F | GGTGGTTTCTGGGGTGAC |
|  | JVHEV R | AGGGGTTGGTTGGATGAA |
|  | JVHEV P | FAM-TGATTCTCAGCCCTTCGC-MGB |
| General HEV (RdRp) | HEV-cs F | TCGCGCATCACMTTYTTCCARAA |
|  | HEV-cas R | GCCATGTTCCAGACDGTRTTCCA |
|  | HEV-csn F | TGTGCTCTGTTTGGCCCNTGGTTYCDG |
|  | HEV-casn R | CCAGGCTCACCRGARTGYTTCTTCCA |
| General HEV (ORF1/2/3 junction) | HEV-4592 F | CCTGGCACCCTCCTTTGGAA |
|  | HEV-5157 R | CGCATGGTGATCCCATGGGC |
|  | HEV-4613 F | TGGAAGAAGCATTCTGGTGAGCC |
